# Bioactive Solution-Blown Polycaprolactone/Gelatin Nanofibers Loaded with *Pistacia lentiscus* Essential Oil: Toward Sustainable and Functional Food Packaging

**DOI:** 10.3390/polym18121511

**Published:** 2026-06-17

**Authors:** Ghizlane Akhouy, Nurcan Dogan, Ali Toptas, Manal Zefzoufi, Rabiaa Fdil, Faissal Aziz, Yasin Akgul, Islam Shyha

**Affiliations:** 1 Water Sciences, Microbial Biotechnologies and Natural Resources Sustainability Laboratory, Faculty of Sciences Semlalia, Cadi Ayyad University, Marrakech 40000, Morocco; ghizlaneakhouy86@gmail.com (G.A.); f.aziz@uca.ac.ma (F.A.); 2 National Center for Research and Studies on Water and Energy, Cadi Ayyad University, Marrakech 40000, Morocco; 3 Department of Food Technology, Bogazliyan Vocational School, Yozgat Bozok University, 66100 Yozgat, Türkiye; nurcan.dogan@yobu.edu.tr; 4 TEMAG Labs, Faculty of Textile Tech and Design, Istanbul Technical University, 34467 Istanbul, Türkiye; atoptas34@gmail.com; 5 Bioorganic Chemistry Team, Faculty of Science, Chouaïb Doukkali University, El Jadida 24000, Morocco; zefzoufi.manal1994@gmail.com (M.Z.); fdilrabia@gmail.com (R.F.); 6 Department of Biomedical Engineering, Faculty of Engineering and Natural Sciences, Karabuk University, 78050 Karabuk, Türkiye; 7 Department of Industrial Engineering, College of Engineering, University of Business and Technology, Jedah 110200, Saudi Arabia; 8 School of Computing Engineering and the Built Environment, Edinburgh Napier University, Edinburgh EH11 4BN, UK

**Keywords:** active packaging, bioactive, nanofibrous, essential oil, storage, Kashar cheese, polycaprolactone, gelatin

## Abstract

Polymer-based active packaging systems incorporating natural bioactive agents have attracted growing interest as eco-friendly alternatives to traditional food packaging materials. In this study, *Pistacia lentiscus* essential oil (PLEO) was incorporated into PCL/gelatin nanofibrous mats fabricated via solution blow spinning (SBS) to develop multifunctional and biodegradable active packaging materials. Neat PCL, gelatin-blended PCL (PCL–G) and PCL–G mats containing 5, 10 and 20 wt.% PLEO were produced and thoroughly analyzed for their morphological, chemical and functional characteristics. Morphological investigation revealed a smooth, bead-free fibrous structure in all samples. The average fiber diameter (AFD) increased from 239 nm to 320 nm with the addition of gelatin to the PCL matrix, while the incorporation of different concentrations of PLEO caused only minor changes. The results showed that as the concentration of PLEO increased, the antioxidant activity of the nanofibrous mats also increased. This enhancement is potentially linked to the rich content of bioactive molecules such as β-pinene, terpineol and verbenol. The 2,2-diphenyl-1-picrylhydrazyl scavenging activity improved from 6.4% (PCL) to 60% (PCL–G–20PLEO), and ABTS activity rose from 8.7% to 72%. In addition, antimicrobial evaluation showed inhibition zones of 12.5 mm against *Escherichia coli* and 14.2 mm against *Staphylococcus aureus* for the PCL–G–20PLEO nanofibrous mats. In 14-day storage tests on Kashar cheese, PCL–G–10PLEO and PCL–G–20PLEO mats reduced microbial counts by more than 2 log units compared with the control and effectively slowed yeast and mold growth. These findings confirm the potential of the PCL–G–PLEO nanofibrous mat as novel active packaging materials for preserving dairy products such as Kashar cheese.

## 1. Introduction

Active food packaging is an emerging and effective strategy that improves the safety, freshness, and quality of food products while extending their shelf life [1]. In contrast to conventional preservation approaches, where active substances are directly incorporated into food matrices, active packaging systems enable the incorporation of bioactive compounds into the packaging material, allowing their controlled release over time. This approach enhances the efficiency of active agents and reduces undesirable interactions with food components [2]. Despite these advantages, commonly used petroleum-based packaging materials such as polyvinyl chloride (PVC), polyethylene (LDPE), polypropylene (PP) and polyethylene terephthalate (PET) pose significant environmental concerns due to their non-biodegradable nature, highlighting the need for sustainable alternatives in food packaging applications [3,4].

Biodegradable materials such as cellulose [5], zein [6] and starch [7] have been widely investigated for use in fibrous mats for food packaging applications. Among these, gelatin is a protein-based polymer extensively used in food packaging, tissue engineering, and biomedical applications due to its biocompatibility and film-forming ability [8]. However, gelatin suffers from its high hydrophilicity and poor mechanical properties, which limit its use in food packaging applications [9]. To overcome these limitations, inorganic nanoparticles, synthetic polymers, and crosslinking have been employed to improve the properties of gelatin-based fibers [10].

Poly(ε-caprolactone) (PCL) is an FDA-approved semi-crystalline aliphatic polyester with excellent mechanical properties, high biocompatibility and biodegradability [3]. PCL is also known for its excellent spinnability and loading capacity, making it a suitable matrix for the incorporation of bioactive compounds in nanofibrous systems. For example, Bahramian et al. reported the development of PCL-based nanofibrous films incorporating lemon beebrush (*Aloysia citriodora*) essential oil-loaded chromium (III) metal–organic framework to extend the shelf life of red meat [11]. Similarly, Thinakaran et al. reported antibacterial PCL nanofibers functionalized with silver nanoparticles using centrifugal spinning [12]. Furthermore, PCL has been reported to act as an effective carrier matrix for natural polymers, improving their processability and structural stability. In this context, blending gelatin with PCL provides a synergistic approach to improve hydrophilicity and functional properties, while PCL contributes to mechanical strength and structural stability [13]. Previous studies have shown that the incorporation of gelatin into PCL matrices increases fiber diameter (from 86.184 ± 20.984 nm to 234.172 ± 98.234 nm) while significantly reducing the water contact angle (from 100.1 ± 3.16° to 55.5 ± 2.10°), indicating improved wettability [14]. Compared with neat PCL nanofibers, this improved hydrophilicity is advantageous for active food packaging applications, as it can promote better moisture interaction. Moreover, the fiber diameter remained within the nanoscale range commonly reported for effective food packaging, ensuring a high surface area and efficient bioactive activity.

Nanofibrous mats have attracted considerable attention in active food packaging due to their high surface area, porous structure, and strong ability to encapsulate and deliver bioactive compounds. Various techniques can be used to produce nanofibrous mats from polymeric solutions. Among them, electrospinning is a well-known technique that allows the formation of fibrous membranes with nanoscale diameters [15]. However, its reliance on high-voltage electric fields and relatively low production rates limits its scalability for industrial applications. In this regard, solution blow spinning (SBS) has emerged as a promising alternative technique that utilizes high-velocity airflow instead of electrical forces to generate fibers [16]. Compared to electrospinning, SBS offers several advantages, including reduced process constraints and significantly higher production rates, which can be up to ten times greater [17]. These advantages make SBS particularly suitable for the large-scale and cost-effective production of nanofibrous materials while also enabling the efficient incorporation and controlled delivery of bioactive compounds in active food packaging applications.

Among the various bioactive compounds incorporated into packaging systems, plant extracts and essential oils have gained significant attention due to their natural antibacterial and antioxidant properties [18]. *Pistacia lentiscus* (PL), a plant belonging to the Anacardiaceae family, is rich in terpenoids and polyphenolic compounds, which contribute to its strong antioxidant and antimicrobial activities [19]. The essential oil derived from this plant (PLEO) has demonstrated remarkable free radical scavenging capacity (125.5 μg/mL) and significant antibacterial effects against various foodborne pathogens, including *Escherichia coli* and *Staphylococcus aureus* [20]. Compared to other essential oils, such as thyme, rosemary, cinnamon, and lavender, which are widely incorporated into active food packaging [21], PLEO remains less explored, particularly in nanofibrous packaging materials. Despite its promising bioactivity, the application of PLEO in food preservation remains challenging due to its rapid degradation and uncontrolled release.

In this context, nanofibrous structures provide a versatile platform for the encapsulation and controlled release of volatile bioactive compounds. The high surface area and porous architecture of nanofibers enable efficient loading and sustained diffusion of essential oils, thereby enhancing their functional performance. Accordingly, the incorporation of PLEO into PCL–gelatin nanofibrous mats is expected to enhance the stability and functional performance of these bioactive compounds, improving their effectiveness in food preservation.

In this study, gelatin and PCL were selected as the polymer matrix due to their complementary physicochemical properties. On one hand, gelatin offers excellent biocompatibility and biodegradability. On the other hand, PCL contributes mechanical stability, flexibility, and enhanced resistance to moisture. Furthermore, *Pistacia lentiscus* essential oil was incorporated as a natural source of antioxidant and antimicrobial compounds. Although essential oils are low-viscosity materials, their incorporation into polymer solutions followed by solution blow spinning enables their encapsulation within the fibrous network. This approach can stabilize volatile compounds while enabling their controlled release during food preservation.

The aim of this study is to develop novel active food packaging based on PCL–G–PLEO nanofibers produced using the SBS technique. The morphological, physicochemical, barrier, antioxidant and antimicrobial properties of the developed nanofibers were systematically investigated, and the influence of PLEO concentration on structural and functional performance was evaluated. Furthermore, the preservation efficiency of the developed materials was assessed using Kashar cheese as a real food model. Kashar cheese was selected because it is a widely consumed semi-hard dairy product with a relatively high moisture content and a nutrient-rich composition that can support the growth of spoilage and pathogenic microorganisms during refrigerated storage. These characteristics make it a suitable model system for evaluating the effectiveness of active packaging materials under realistic storage conditions. The novelty of this work lies in the use of *Pistacia lentiscus* essential oil in SBS-produced PCL/gelatin nanofibrous mats and the evaluation of their effectiveness in a real food preservation application. To the best of our knowledge, this study is among the first to combine solution blow spinning (SBS) with the incorporation of PLEO into PCL–gelatin nanofibrous structures for active food packaging applications and to validate their performance in a real food system.

## 2. Materials and Methods

### 2.1. Materials

Polycaprolactone (PCL, Mn ≈ 80,000, density 1.145 g/mL) was supplied by Sigma-Aldrich (Gillingham, UK). Gelatin (250–270 Bloom, type B), produced from bovine skin, was supplied in powder form by Halavet Gida LLC (Istanbul, Türkiye). Formic acid (85%, 1.20 g/cm^3^) and acetic acid (80% purity, 1.08 g/cm^3^) were supplied by Tekkim (Bursa, Türkiye).

### 2.2. Plant Material

Fresh aerial parts of *Pistacia lentiscus* were collected from the Ourika Valley, located in the Tensift Al-Haouz region, Marrakech, Morocco. The collection area is situated in the foothills of the High Atlas Mountains at an altitude ranging between 900 and 1500 m above sea level. The region is characterized by a semi-arid Mediterranean climate with hot, dry summers and mild winters.

The samples were washed under running tap water, shade-dried at room temperature, and subsequently ground into powder. A voucher specimen of this species has been stored in the Herbarium of the Botany Department at the Scientific Institute of Rabat (Morocco), under voucher code RAB30.

### 2.3. Extraction of PLEO

PLEO was extracted from the aerial parts of *Pistacia lentiscus* by hydrodistillation using a Clevenger-type apparatus for 4 h. The collected oil was dried over anhydrous sodium sulfate and stored in airtight containers at 4 °C in the dark until further use.

### 2.4. Fabrication Methods

#### 2.4.1. Preparation of Solutions

Five polymeric solutions were formulated to produce nanofibrous mats. Table 1 summarizes the composition of the prepared solutions. Preliminary experiments demonstrated that a total polymer concentration of 15 wt.% provided sufficient viscosity for stable fiber formation during solution blow spinning, resulting in smooth and bead-free nanofibers. The acetic acid/formic acid co-solvents were used at a ratio of 80/20, and the solutions were stirred for 6 h to ensure homogeneity. In addition, varying quantities of PLEO were introduced into PCL-G solutions, specifically 5, 10, and 20 wt.% relative to the total polymer weight, and each mixture was stirred for an additional 30 min at room temperature.

#### 2.4.2. Production of Nanofibrous Mats

**Figure 1:**
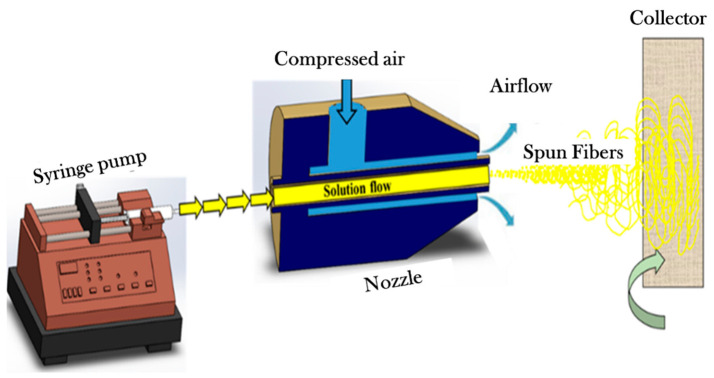
Schematic diagram of SBS.

Nanofibers were produced using an SBS system (Aerospinner L1.0, AREKA Group, Beyoğlu, Turkey) equipped with a concentric nozzle configuration. The polymer solution was delivered through an 18-gauge needle using an NE-1000 syringe pump (New Era Pump Systems, Farmingdale, NY, USA) (Figure 1). The flow rate was maintained at 10 mL/h, while compressed air at 1 bar was supplied through the outer nozzle to generate fibers. The fibers were collected on a rotating collector positioned 45 cm from the nozzle, using a spunbond polypropylene substrate. All SBS experiments were conducted under standard laboratory conditions at room temperature, with relative humidity varying between approximately 40% and 60%.

### 2.5. Characterization of Fibrous Mats

GC–MS analysis of PLEO was performed using a Thermo 8000 gas chromatograph (Thermo Fisher, Waltham, MA, USA) equipped with a non-polar Hewlett–Packard OV-17 capillary column (25 m × 0.25 mm i.d., 0.25 μm film thickness). The column oven temperature was initially set to 60 °C and maintained for 6 min, followed by a 5 °C/min increase to 150 °C and then held for an additional 10 min. The total run time was 34 min. Helium was used as the carrier gas at a constant flow rate of 2 mL/min in splitless injection mode to maximize analyte transfer onto the column. The injector temperature was maintained at 225 °C, while the detector temperature was set to 250 °C to ensure optimal volatilization and detection of the essential oil components.

The FTIR spectra of the nanofibrous mats were obtained using a Bruker ALPHA FTIR spectrometer (Bruker, Billerica, MA, USA). A total of 24 scans were recorded in the range of 400–4000 cm^−1^.

The morphology of the nanofibers was examined using a Carl Zeiss Ultra Plus Field Emission Scanning Electron Microscope (FE-SEM) (Carl Zeiss, Oberkochen, Germany). The average fiber diameters (AFD) and their distributions were determined from SEM images using ImageJ 1.54g software. For this purpose, at least 100 measurements were performed in randomly selected regions.

The thickness of the nanofibrous mats was measured using a digital micrometer with an accuracy of ±1 μm.

The air permeability of the produced nanofibers was evaluated in accordance with ASTM D737 [22] using a Prowhite Air Test II device (PRO-SER, Istanbul, Türkiye). A sample area of 38 cm^2^ was tested under an air pressure of 125 Pa. The experiments were conducted at room temperature.

The tensile properties of all samples were determined using uniaxial tensile testing with a crosshead speed of 1 mm/min. All samples were cut into rectangles with dimensions of 100 × 20 mm^2^ and horizontally mounted on gripping units, leaving a 40 mm gauge length.

Surface wettability was evaluated with an optical tensiometer (Theta Lite, Biolin Scientific, Västra Frölunda, Sweden) via the sessile drop method with 0.0085 mL of distilled water. Samples were cut into 50 × 50 mm squares.

X-ray diffraction (XRD) analysis was performed using a Rigaku Ultima IV diffractometer (Rigaku, Tokyo, Japan) equipped with Cu Kα radiation (λ = 0.154 nm). The measurements were conducted at 40 kV and 40 mA over a 2θ range of 10–90° with a step size of 0.03° at room temperature.

The antioxidant potential of the nanofiber mats was evaluated using DPPH and ABTS radical scavenging assays with a UV–Vis spectrophotometer (Optizen, Mecasys, Daejeon, Republic of Korea). Table 2 summarizes the experimental parameters used to evaluate the antioxidant activity of the nanofibrous mats. Approximately 4 cm^2^ of nanofibrous mat, based on the average size of the prepared samples, was used.

The radical scavenging activity (%) for both assays was calculated using standard equations, where A_0_ represents the control absorbance and A represents the absorbance in the presence of the extract. All results were expressed as percent inhibition (mean ± standard deviation) from triplicate measurements.

**Figure 2:**
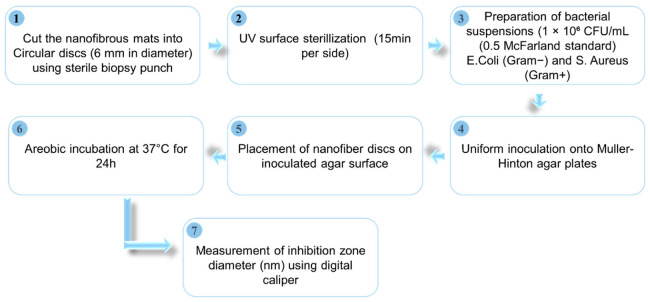
Schematic representation of the antibacterial testing protocol applied to nanofibers.

Bacteria capable of causing foodborne illness, such as Gram-negative *E. coli* and Gram-positive *S. aureus*, were tested to assess the efficacy of nanofibrous mats against them. Figure 2 represents the antibacterial testing protocol applied to the produced nanofibers.

A control sample (PCL–G without PLEO) was also tested under identical conditions for comparison. Each condition was evaluated in triplicate, and the results were reported as mean ± standard deviation.

### 2.6. Packaging Tests

The preservation performance of the PLEO-loaded nanofibrous mats was evaluated using Kashar cheese as a real food model. Cheese blocks were cut into uniform pieces measuring approximately 30 × 30 × 4 mm (see Figure S1), after which each piece was separately inoculated with either *E. coli* or *S. aureus*. These two strains were selected as foodborne pathogens commonly associated with fresh produce. Fresh bacterial suspensions were prepared at 1 × 10^6^ CFU/mL (0.5 McFarland standard), and 100 µL of each suspension was evenly spread onto the surface of the cheese samples to ensure consistent contamination.

Prior to packaging, the nanofiber mats were sterilized under UV light for 15 min on each side. The inoculated cheese pieces were then wrapped with a double layer of the sterilized nanofibers to ensure full surface coverage, while unwrapped cheese samples served as the control group. All samples were individually placed in sterile glass Petri dishes and stored at 4 ± 1 °C.

Microbiological analyses were performed on days 0, 3, 7, and 14 of storage. At each sampling time, cheese samples were aseptically homogenized in sterile diluent, serially diluted, and plated onto selective media for enumeration of *E. coli* (VRBA), *S. aureus* (Baird–Parker agar), total viable counts (TVC, Plate Count Agar), and yeast and molds (potato dextrose agar). Plates were incubated under appropriate conditions for each microbial group, and the results were expressed as log CFU/g cheese.

## 3. Results and Discussion

### 3.1. Identification of EO Composition

Essential oils are rich in bioactive compounds such as monoterpene hydrocarbons, oxygenated monoterpenes and sesquiterpenes, which are well known for their strong antioxidant and antibacterial properties (Bhavaniramya et al., 2019) [23]. The chemical composition of PLEO is shown in Table 3. A total of 13 molecules identified by GC-MS represented 93.69% of the oil content. The composition was dominated by monoterpene hydrocarbons (83.06%). Among the identified constituents, β-pinene (35.25%) was the most abundant component, followed by terpineol (14.68%), verbenol (9.22%), terpinene (8.13%), limonene (6.47%), carene (5.34%), and p-cymene (5.11%).

Previous studies have demonstrated that PLEO exhibits strong antioxidant activity, as indicated by its low IC_50_ value (70.88 μg/mL) in DPPH assays. In addition, it shows considerable antibacterial activity, with inhibition zones of 14 mm and 20 mm against *Listeria* and *Bacillus*, respectively, as well as activity against *E. coli*. These results highlight the potential of PLEO as a natural bioactive agent for active food packaging applications [24].

### 3.2. FTIR Analysis

**Figure 3:**
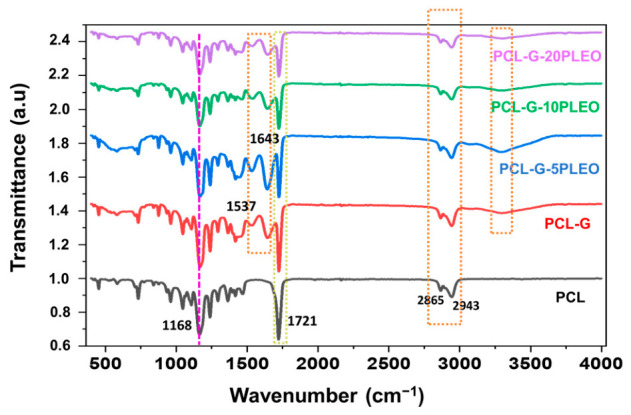
FTIR analysis of nanofibrous mats.

The FTIR spectra of PCL, PCL–G, and PCL–G–PLEO nanofibrous mats are shown in Figure 3. The PCL nanofibers revealed two prominent peaks at a wavelength of 2943 and 2865 cm^−1^, corresponding to the stretching vibrations of aliphatic C–H groups. The characteristic peaks observed at 1721 cm^−1^ and 1170 cm^−1^ correspond to the carbonyl (C=O) stretching of ester groups and symmetric C–O–C stretching, respectively [25]. For the PCL–G samples, additional peaks appeared at 1643 cm^−1^ (amide I, C=O stretching), 1537 cm^−1^ (amide II, N–H bending and C–N stretching), and 1417 cm^−1^ (CH_2_ bending), confirming the successful incorporation of gelatin into the polymer matrix [25,26]. The incorporation of PLEO, even at a relatively high concentration (20 wt.%), did not introduce any new characteristic peaks or cause noticeable shifts in the FTIR spectra compared to the PCL–G sample, suggesting the absence of significant chemical interactions and indicating that PLEO was incorporated mainly through physical encapsulation. The same findings were reported in our previous studies, where no significant differences in band positions were observed after the incorporation of *Origanum elongatum* essential oil to G-Ch-PVA nanofibers and *Cedrus atlantica* essential oil to G-Ch-PA6 [27,28]. Hani et al. (2017) also reported that there was no significant difference in band position after the addition of Moringa oleifera bioactive extract into gelatin nanofibers [29]. Since strong chemical interactions between PLEO and the functional groups of the polymeric matrix could disrupt the nanofiber structure and lead to uncontrolled release, the physical encapsulation of PLEO is beneficial for food packaging applications. This can preserve the integrity of the nanofibrous mat structure and promote a more controlled release of bioactive compounds, thereby improving the functional performance of the packaging material.

### 3.3. X-Ray Diffraction

**Figure 4:**
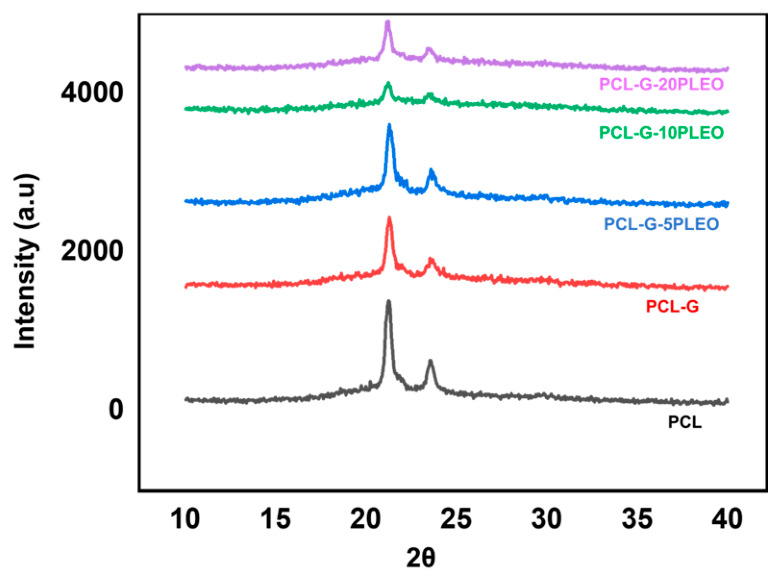
X-ray diffraction patterns of nanofibrous mats.

Figure 4 presents the XRD patterns of the PCL, PCL–G, PCL–G–5PLEO, PCL–G–10PLEO and PCL–G–20PLEO nanofibrous mats. PCL nanofibers exhibited two characteristic peaks at 2θ = 21.11° and 23.99°, which correspond to the (110) and (200) planes, respectively [30]. The decrease in the intensity of the characteristic PCL diffraction peaks suggests a reduction in crystalline order following gelatin and PLEO incorporation. However, the degree of crystallinity was not quantitatively determined in the present study. This can be explained by a reduction in crystallinity attributable to the amorphous nature of gelatin [31]. The incorporation of PLEO into the PCL-G matrix further decreased the intensity of these crystalline peaks. Furthermore, an even more pronounced reduction was observed at 20 wt.% PLEO, which can be related to the plasticizing effect of the essential oil. The essential oil interferes with the polymeric chains, reducing crystalline order [32]. Thus, the XRD results are consistent with the FTIR findings, indicating that PLEO incorporation modifies the structural organization of the PCL–G matrix by decreasing crystallinity without the formation of new chemical bonds.

### 3.4. Morphologies of Nanofibrous Mats

**Figure 5:**
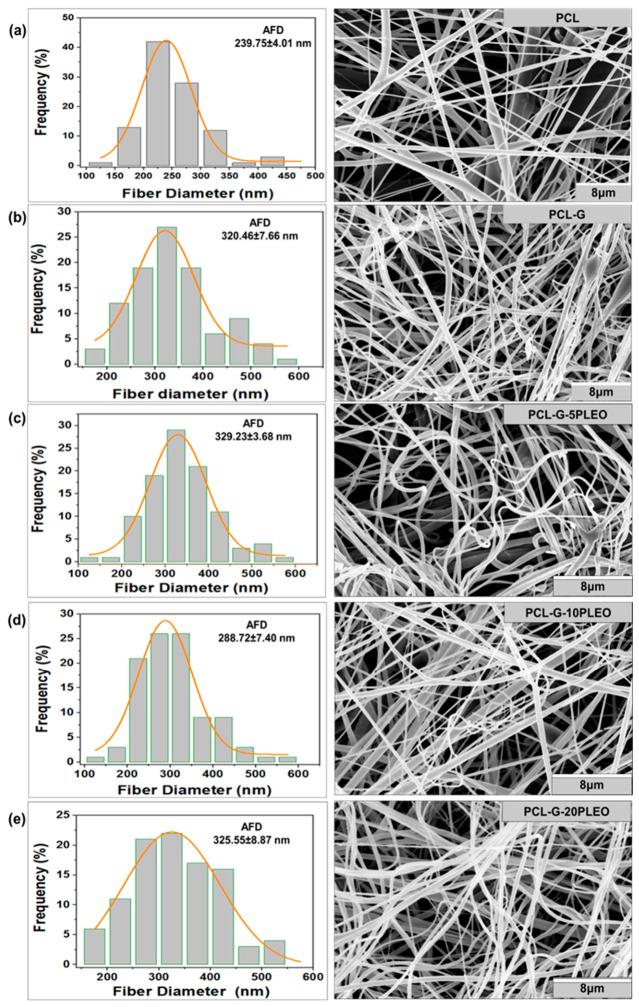
SEM images of (**a**) PCL, (**b**) PCL-G, (**c**) PCL-G-5PLE, (**d**) PCL-G-10PLEO, (**e**) PCL-G-20PLEO nanofibrous mats and their AFD distributions.

SEM was used to study the morphology of the produced nanofiber mats, and the SEM images, along with the fiber diameter distributions of the samples, are shown in Figure 5, SEM observations indicated that all formulations produced smooth, continuous, and bead-free fibers. No visible surface defects, cracks, or pronounced roughness changes were detected following gelatin or PLEO incorporation. The most evident morphological effect was the change in average fiber diameter, while the overall surface appearance remained largely unchanged within the magnification range examined. This result indicates the absence of PLEO aggregation on the fiber surface and suggest that incorporating PLEO into the PCL-G solution did not affect the fiber morphology. The AFD of pure PCL nanofibers was 239.75 ± 4.01 nm, which increased to 320.46 ± 7.66 nm upon gelatin incorporation, consistent with the higher apparent viscosity qualitatively observed during solution preparation due to the presence of gelatin. With the incorporation of PLEO, a progressive increase in fiber diameter was observed. At 5 wt.% PLEO content, the AFD reached about 329.23 ± 3.68 nm. Similar results were corroborated by Unalan et al., who also observed an increase in fiber diameter with the addition of clove essential oil compared to PCL–G nanofibers [33]. This variation in fiber diameter could be attributed to changes in the viscosity and evaporation rate of the polymer solution [34]. Furthermore, a slight reduction in diameter was observed at 10 wt.% PLEO, reaching 288.72 ± 7.40 nm. The decrease in fiber diameter can be explained by the plasticizing effect of the essential oil [35]. In addition, the good miscibility between the two hydrophobic materials, namely the essential oil and PCL, likely improved the solution’s homogeneity. Since PCL is a hydrophobic polymer with good elasticity, it blends well with the hydrophobic essential oil, resulting in thinner and more uniform fibers [36].

However, at 20 wt.% PLEO, a notable increase was observed, with the fibers measuring an average diameter of 325.55 ± 8.87 nm. This can be attributed to the hydrophobic nature of the essential oil and its influence on the physicochemical properties of the polymeric solution. At high concentrations, hydrophobic oil droplets tend to aggregate within the polymeric solution, which leads to increased phase heterogeneity and reduced compatibility between the oil and the polymer matrix.

### 3.5. Water Contact Angles of Nanofibrous Mats

The average contact angle values of the nanofibrous mats are presented in Table 4. The hydrophobic nature of PCL is reflected by the high contact angle of 132.72°. Furthermore, the hydrophilicity of PCL nanofibers was enhanced with the addition of G. Although PLEO is hydrophobic, at low concentrations (5 and 10 wt.%), it modifies the phase behavior of the PCL-G matrix, thereby promoting the migration of hydrophilic O–H groups toward the surface. This leads to an enrichment of polar functional groups at the fiber surface and a significant reduction in the contact angle. However, at 20 wt.% PLEO, the fiber surface is dominated by the hydrophobic groups of the essential oil, leading to an increase in hydrophobicity to 134.05°.

### 3.6. Air Permeability and Mechanical Properties of Nanofibrous Mats

Air permeability plays a vital role in food packaging materials. By controlling the transmission of gases such as oxygen, CO_2_ and water vapor, it ensures food safety, extends shelf life and maintains product quality. Several studies have confirmed that air permeability is directly influenced by factors including fiber diameter, porosity, thickness, and packing density of nanofibrous mats [37]. The air permeability and thickness values of the produced nanofibrous mats are presented in Table 5. The thickness of the samples increased from 133 ± 10 µm for neat PCL nanofibers to 191 ± 23 µm for PCL-G-20PLEO nanofibers. This increase can be attributed to the incorporation of gelatin and PLEO, which resulted in larger fiber diameters and greater material deposition during the SBS process. However, despite the gradual increase in thickness, air permeability did not show a direct inverse correlation with thickness, indicating that structural parameters such as fiber diameter distribution, pore architecture, and packing density also played important roles in determining air transport through the nanofibrous mats.

The PCL nanofibrous mat exhibited the highest air permeability, at about 14 mm/s, indicating a relatively porous structure. With the addition of G to the PCL matrix, the air permeability decreased to 9 mm/s. A similar effect was reported for PCL-G nanofibers, where air permeability decreased as the PCL content in the polymeric matrix was reduced [38]. When 5 wt.% PLEO was added, the air permeability increased to 12.66 mm/s. As the PLEO concentration increased to 10 wt.%, air permeability dropped significantly to 6 mm/s. The lower air permeability of the PCL-G-10PLEO sample can be attributed to its smaller average fiber diameter, which promotes the formation of a denser fibrous network with reduced pore size. The resulting decrease in inter-fiber voids limits air transport through the structure and leads to lower permeability values. Therefore, the PCL-G-10 wt.% PLEO sample exhibits lower breathability than the other samples, making it beneficial for active food packaging applications.

The mechanical properties of the nanofibrous mats are presented in Table 6. Pure PCL nanofibrous mats exhibited the highest tensile strength (0.80 MPa) and elongation at break (28.88%). The incorporation of gelatin resulted in a reduction in both tensile strength and elongation, with values decreasing to 0.52 MPa and 23.17%, respectively. Furthermore, the addition of PLEO caused a concentration-dependent decline in mechanical performance. The tensile strength decreased from 0.47 MPa for PCL-G-5PLEO to 0.28 MPa for PCL-G-20PLEO, while the elongation at break decreased from 18.00% to 8.37%. This behavior may be attributed to the disruption of fiber continuity and intermolecular interactions within the polymer matrix caused by the incorporation of increasing amounts of essential oil. Similar reductions in mechanical properties following the addition of essential oils have been reported previously and are generally associated with phase separation, structural heterogeneity, and the formation of weak points within the fibrous network [27,28,37]. Nevertheless, all formulations maintained sufficient structural integrity for handling and potential active food packaging applications.

### 3.7. Antioxidant Activity of Nanofibrous Mats

The antioxidant performance of the nanofibrous mats was evaluated using DPPH and ABTS radical scavenging assays, and the results are presented in Table 6. Both assays revealed that the increase in radical scavenging activity depended directly on the concentration of PLEO incorporated into the polymeric matrix. PCL and PCL–G mats showed minimal antioxidant activity, with DPPH and ABTS inhibition values below 16%. This could be explained by the absence of bioactive molecules, including phenolic or terpene functional groups capable of donating hydrogen atoms or electrons. In contrast, a significant increase in radical scavenging capacity was observed, reaching 60.0 ± 2.0% and 72.0 ± 3.0% inhibition in the DPPH and ABTS assays, respectively, for the PCL–G–20PLEO sample. This enhancement indicates that the bioactive compounds in PLEO were efficiently entrapped within the nanofiber matrix and remained reactive toward free radicals. This significant antioxidant activity of PLEO-loaded mats is attributed to the high contents of β-pinene (35.25%), terpineol (14.68%), verbenol (9.22%), terpinene (8.13%), and limonene (6.47%), as determined by GC–MS analysis (Table 2). The presence of allylic and hydroxyl functional groups, capable of donating hydrogen atoms and stabilizing unpaired electrons, in the structure of these molecules ensures strong radical-scavenging performance [19,20]. β-pinene and terpineol have been reported to exhibit high reactivity toward both DPPH• and ABTS•^+^ radicals, which helps explain the greater efficiency observed in ABTS assays, where electron-transfer mechanisms predominate [39]. Moreover, the synergistic effects of verbenol and p-cymene enhance scavenging potential through resonance-stabilized radical intermediates [40]. The observed increase in antioxidant capacity at higher PLEO concentrations suggests that PLEO components were protected within the nanofiber network. This phenomenon allosws a sustained release and stable reactivity. The release of PLEO from the nanofibrous structure is expected to occur mainly through diffusion from the physically encapsulated polymer matrix. The hydrophilic nature of gelatin promotes moisture uptake and swelling under food storage conditions, facilitating the migration of bioactive compounds. In contrast, the hydrophobic PCL phase provides structural integrity and slows the rapid volatilization of essential oil components, resulting in a more sustained release profile. Furthermore, the high surface area and porous architecture of the nanofibers enhance mass transfer, allowing the gradual release of antioxidant and antimicrobial compounds during storage. Comparable trends have been reported in other essential-oil-based nanofibers. Unalan et al. [33] observed that the addition of clove essential oil to PCL–gelatin nanofibers increased DPPH scavenging up to 52.8%, while Doğan et al. [41] achieved 54.5% DPPH and 68.0% ABTS inhibition using lavender oil-loaded nanofibers. These results corroborate the finding that essential oils with high monoterpenoid content could effectively improve the antioxidant activity of nanofiber mats. Although the increase was modest, the difference between 10 wt.% and 20 wt.% PLEO loading was significant. This behavior arises from partial saturation of the polymer network and diffusion constraints that limit the exposure of active sites to radicals. Mendes et al. [42] observed a similar trend for peppermint essential oil-loaded PLA nanofibers. Overall, the results confirm that PLEO incorporation into PCL-G nanofibers substantially enhance their antioxidant properties through the synergistic activity of monoterpene constituents. In addition, the encapsulation of PLEO acts as a protective barrier against environmental factors that are known to degrade terpenoids. These findings align with the hypothesis that the antioxidant potential of essential-oil-loaded nanofibers depends not only on the total phenolic or terpene content but also on the effective diffusion and stabilization of these compounds within the polymeric structure, which directly governs their ability to neutralize reactive species.

### 3.8. Antimicrobial Activity of Nanofibrous Mats

The antibacterial activity of the nanofibrous mats was assessed against *E. coli* and *S. aureus* using the agar disk diffusion method, with the results summarized in Table 7. Negligible inhibitory activity was observed for PCL and PCL-G nanofibers, confirming that both samples lack inherent antibacterial functionality. In contrast, the incorporation of PLEO resulted in a significant enhancement in antibacterial performance. Here, the inhibition zones increased progressively alongside oil concentration. The PCL-G-20PLEO sample exhibited the largest inhibition halos, measuring 12.5 ± 0.6 mm for *E. coli* and 14.2 ± 0.7 mm for *S. aureus*. This indicates that the bioactive molecules of PLEO were effectively released from the nanofiber matrix and preserved their biological activity during testing. The pronounced antimicrobial effect of PLEO-loaded mats can be attributed to the synergistic interaction of their major monoterpene constituents, particularly β-pinene (35.25%), terpineol (14.68%), verbenol (9.22%), terpinene (8.13%), and limonene (6.47%). These compounds are well documented for their membrane-disrupting and protein-denaturing capabilities, which contribute to bacterial cell lysis and leakage of cytoplasmic contents [19,20]. Specifically, β-pinene and terpineol act as hydrophobic agents that integrate into the lipid bilayer and increase membrane permeability, causing structural disorganization [37]. On the other hand, limonene and p-cymene enhance this mechanism by altering membrane fluidity and promoting ion efflux, while verbenol contributes to oxidative stress within microbial cells through reactive oxygen species generation [39]. As expected, *S. aureus* showed greater sensitivity to the PLEO-loaded mats than *E. coli*, which can be explained by differences in cell wall structure. The outer lipopolysaccharide layer of *E. coli* serves as an additional barrier, restricting the diffusion of hydrophobic compounds [37,41]. However, the single-layered peptidoglycan structure of *S. aureus* allows for faster penetration of PLEO components, leading to stronger inhibitory effects. Similar behavior was observed in other studies using essential oil-based nanofibers. Akhouy et al. [27] reported that electroblown gelatin/chitosan nanofibers containing *Origanum elongatum* essential oil exhibited inhibition zones of 13.8 mm against *S. aureus* and 9.2 mm against *E. coli*, values closely matching the results obtained in this study. Furthermore, at higher PLEO concentrations, antibacterial activity increased from 7.2 mm to 12.5 mm for *E. coli* and from 9.1 mm to 14.2 mm for *S. aureus*. This is consistent with the increased availability of volatile terpenoids at the nanofiber surface, which facilitates sustained diffusion into the microbial environment. Nevertheless, the difference between 10 wt.% and 20 wt.% PLEO loadings, although statistically significant (*p* < 0.05), was relatively modest. This indicates partial saturation of available polymer binding sites and diffusion limitations, which may occur at high oil contents. Overall, the antimicrobial results confirm that incorporating PLEO into PCL-G nanofibers effectively enhances bacterial inhibition via the release of monoterpenes. These compounds interact synergistically to damage microbial membranes and interfere with cellular metabolism. The strong correlation between the GC–MS composition and antibacterial performance further supports the conclusion that PLEO’s bioactivity is directly responsible for the functional properties of the nanofibrous mats, validating their potential use in active food packaging applications.

### 3.9. Antimicrobial Performance of Nanofibers During Cheese Storage

**Figure 6:**
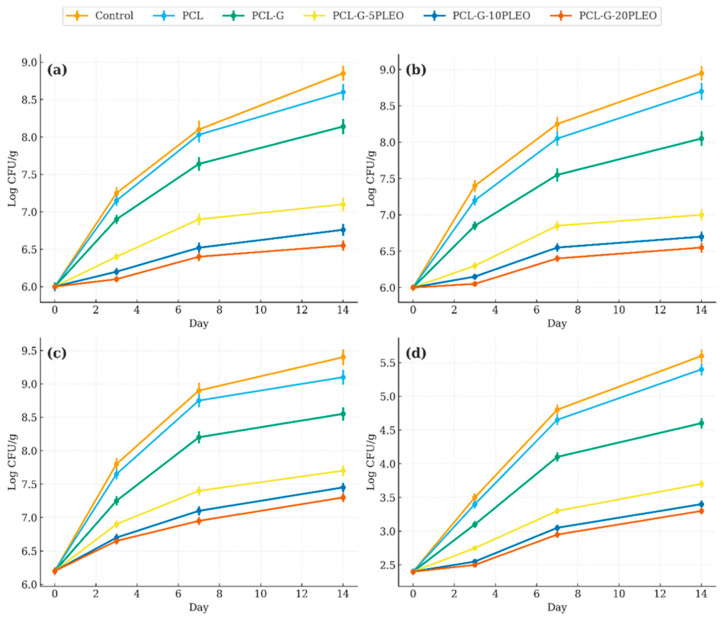
Microbial growth on cheese during 14 days of refrigerated storage. (**a**) *E. coli*, (**b**) *S. aureus*, (**c**) total viable counts (TVC), and (**d**) yeast and mold. Values represent mean ± SD (*n* = 3).

The antimicrobial behavior of nanofiber-wrapped cheese samples during 14 days of refrigerated storage is shown in Figure 6. The results showed a clear concentration-dependent inhibition of *E. coli*, *S. aureus*, TVC, and yeast–mold populations. Throughout storage, the control samples exhibited the fastest microbial growth, reaching 8.85 log CFU/g for *E. coli* and 8.98 log CFU/g for *S. aureus*. This agrees with earlier reports showing that semi-hard cheeses permit rapid proliferation of both Gram-negative and Gram-positive microorganisms under refrigeration [43,44]. The incorporation of PLEO into PCL-G nanofibers markedly reduced microbial growth, with PCL-G-10PLEO and PCL-G-20PLEO limiting *E. coli* counts to 6.76 and 6.55 log CFU/g, respectively, by day 14. The stronger inhibition observed for *S. aureus*, with final counts of 6.70 and 6.55 log CFU/g at 10% and 20% PLEO, reflects the greater sensitivity of Gram-positive cells to hydrophobic terpenoids due to their more permeable peptidoglycan layer. This observation is consistent with previous findings on the antimicrobial potency of monoterpenes such as β-pinene, terpineol, limonene, and caryophyllene [45]. Similar trends were observed in TVC and yeast–mold counts, where PLEO-loaded mats effectively slowed the growth of general spoilage flora, reducing fungal proliferation by more than 2 log units compared to the control. This is in line with studies reporting antifungal effects of terpene-rich essential oils through ergosterol disruption and mitochondrial inhibition [46]. Collectively, the results indicate that PLEO-loaded nanofibers provide effective, broad-spectrum microbial inhibition in cheese, supporting their potential application as active food packaging materials.

## 4. Conclusions

The study focused on using a solution blow spinning method to develop a nanofibrous mat for food packaging applications. The active characteristics such as the antioxidant and antimicrobial functionalities of the package were ensured by incorporating PLEO, which exhibits strong antioxidant and antimicrobial activities. Neat PCL, PCL–G, and PCL–G–5PLEO, PCL–G–10PLEO, and PCL–G–20PLEO nanofibers were successfully produced by SBS. GC-MS analysis of PLEO revealed a high proportion of bioactive molecules, including β-pinene, terpineol, and verbenol. The mean fiber diameters of the produced nanofibers were 239.75 ± 4.01 nm, 320.46 ± 7.66 nm and 325.55 ± 8.87 nm for PCL, PCL–G, and PCL–G–20PLEO samples, respectively. FTIR and XRD analyses revealed that PLEO was physically encapsulated within the polymer matrix without forming chemical bonds, and its incorporation reduced the degree of crystallinity of polymeric chains. In addition, the antioxidant activity of the samples was enhanced by the addition of PLEO into the nanofiber matrix. A remarkable rise in both DPPH and ABTS radical-scavenging percentages was achieved. At 20 wt.% PLEO loading, the DPPH and ABTS radical scavenging activities reached 60% and 72%, respectively. Antimicrobial testing showed that PLEO-enriched nanofibers exhibited strong inhibitory activity, with inhibition zones of 14.2 mm against *S. aureus* and 12.5 mm against *E. coli*. In 14-day storage tests on Kashar cheese, PCL-G-10PLEO and PCL-G-20PLEO mats reduced microbial counts by more than 2 log units compared with the control and effectively slowed yeast and mold growth. Overall, the findings show that PLEO-loaded PCL–G nanofibers offer high potential as sustainable, eco-friendly active packaging materials with enhanced antioxidant and antimicrobial performance. Furthermore, the results highlight the effectiveness of essential oils as natural agents for improving food safety via release from nanofiber-based carrier systems.

## Figures and Tables

**Table 1:** Composition of the prepared polymer solutions.

Sample Code	PCL Concentration (%*w*/*w*)	G Concentration (%*w*/*w*)	Acetic Acid Concentration (%*w*/*w*)	Formic Acid Concentration (%*w*/*w*)	PLEO Concentration (%*w*/*w*)
PCL	15	-	68	17	-
PCL-G	10	5	68	17	-
PCL-G-5PLEO	9.5	4.75	68	17	0.75
PCL-G-10PLEO	9	4.5	68	17	1.5
PCL-G-20PLEO	8	4	68	17	3

**Table 2:** Summary of experimental parameters for antioxidant activity determination of the nanofibrous mats.

Parameter	DPPH Radical Scavenging Assay	ABTS Radical Scavenging Assay
Nanofibrous sample	4 cm^2^	4 cm^2^
Immersion solvent	80% ethanol (*v*/*v*)	80% ethanol (*v*/*v*)
Immersion volume	10 mL	10 mL
Sample preparation conditions	Gentle agitation (200 rpm) for 1 h at ambient temperature in the dark	Gentle agitation (200 rpm) for 1 h at ambient temperature in the dark
Filtration	0.45 µm membrane filter	0.45 µm membrane filter
Radical solution preparation	0.1 mM DPPH solution (prepared in methanol immediately before use)	ABTS•^+^ radicals generated by mixing 7.0 mM ABTS and 2.45 mM potassium persulfate (1:1, *v*/*v*), incubated in dark for 12–16 h
Working solution adjustment	-	Diluted with 80% ethanol to absorbance of 0.70 ± 0.02 AT 734 nm.
Reaction mixture	1.5 mL extract + 1.5 mL DPPH solution	0.20 mL was added to 2.0 mL ABTS•^+^ working solution
Incubation conditions	30 min in the dark	6 min at ambient conditions
Measurement wavelength	517 nm	734 nm

**Table 3:** Composition of PLEO.

No	Component	RI*	%
1	Myrcene	948	3.11
2	Camphene	949	1.04
3	Alpha-phellandrene	964	1.7
4	β-pinene	966	35.25
5	Terpinene	998	8.13
6	Carene	1005	5.34
7	Limonene	1018	6.47
8	p-cymene	1042	5.11
9	Verbenol	1122	9.22
10	Terpineol	1137	14.68
11	Borneol	1138	3.28
12	p-cymenol	1042	0.1
13	Farnesene	1458	0.26
	Total identified compounds		93.69

RI* refers to the retention index used for compound identification in GC-MS analysis.

**Table 4:** Contact angle of nanofibrous mats.

Sample Code	Contact Angle (°)	Standard Deviation
PCL	132.72	0.64
PCL-G	109.91	0.52
PCL-G-5PLEO	54.61	22.65
PCL-G-10PLEO	29.97	8.48
PCL-G-20PLEO	134.05	1

**Table 5:** Air permeability values and tensile test results of the nanofibrous mats.

Sample Code	Air Permeability (mm/s)	Thickness (µm)	Max Strength (MPa)	Max Strain (%)
PCL	14 ± 0.0	133 ± 10	0.80 ± 0.14	28.88 ± 4.2
PCL-G	9 ± 0.0	156 ± 7	0.52 ± 0.12	23.17 ± 4.8
PCL-G-5PLEO	12.66 ± 0.57	160 ± 16	0.47 ± 0.12	18 ± 3.2
PCL-G-10PLEO	6 ± 0.0	183 ± 10	0.34 ± 0.09	14.82 ± 2.8
PCL-G-20PLEO	9 ± 0.0	191 ± 23	0.28 ± 0.08	8.37 ± 2.7

**Table 6:** Antioxidant activities of nanofibrous mats.

Samples	DPPH (%)	ABTS (%)
PCL	6.4 ± 0.5 ^e^	8.7 ± 0.6 ^e^
PCL-G	12.8 ± 0.8 ^d^	15.4 ± 0.9 ^d^
PCL-G-5PLEO	35.7 ± 2.1 ^c^	46.2 ± 2.3 ^c^
PCL-G-10PLEO	56.3 ± 2.8 ^b^	66.8 ± 3.0 ^b^
PCL-G-20PLEO	60.0 ± 2.0 ^a^	72.0 ± 3.0 ^a^

Different superscript letters (a–e) within the same column indicate statistically significant differences (*p* < 0.05).

**Table 7:** Antimicrobial activities of nanofibrous mats.

Samples	*E. coli* (mm)	*S. aureus* (mm)
PCL	0.0 ± 0.0 ^d^	0.0 ± 0.0 ^d^
PCL-G	0.5 ± 0.5 ^d^	1.4 ± 0.6 ^d^
PCL-G-5PLEO	7.2 ± 0.6 ^c^	9.1 ± 0.7 ^c^
PCL-G-10PLEO	11.1 ± 0.7 ^b^	13.0 ± 0.8 ^b^
PCL-G-20PLEO	12.5 ± 0.6 ^a^	14.2 ± 0.7 ^a^

Different superscript letters (a–d) within the same column indicate statistically significant differences (*p* < 0.05).

## Data Availability

The original contributions presented in this study are included in the article/Supplementary Material. Further inquiries can be directed to the corresponding authors.

## Supplementary Materials

The following supporting information can be downloaded at: https://www.mdpi.com/article/10.3390/polym18121511/s1, Figure S1: Cheese samples before and after application of nanofibrous mats. (a) Untreated cheese samples prior to wrapping. (b) Cheese samples after wrapping with nanofibrous mats including the following formulations: Control, PCL, PCL-G, PCL-G-5PLEO, PCL-G-10PLEO, and PCL-G-20PLEO. Each cheese piece was wrapped with a double layer of the corresponding nanofiber film, except the control sample, which remained unwrapped.
