# Supplementary material for: Bioactive Solution-Blown Polycaprolactone/Gelatin Nanofibers Loaded with Pistacia lentiscus Essential Oil: Toward Sustainable and Functional Food Packaging
Source: Polymers (Basel). 2026 Jun 17;18(12):1511. doi: 10.3390/polym18121511 (PMC13307172; doi:10.3390/polym18121511)
Supplement: Supplementary file 1 [file polymers-18-01511-s001.zip › polymers-4364617-supplementary.pdf]

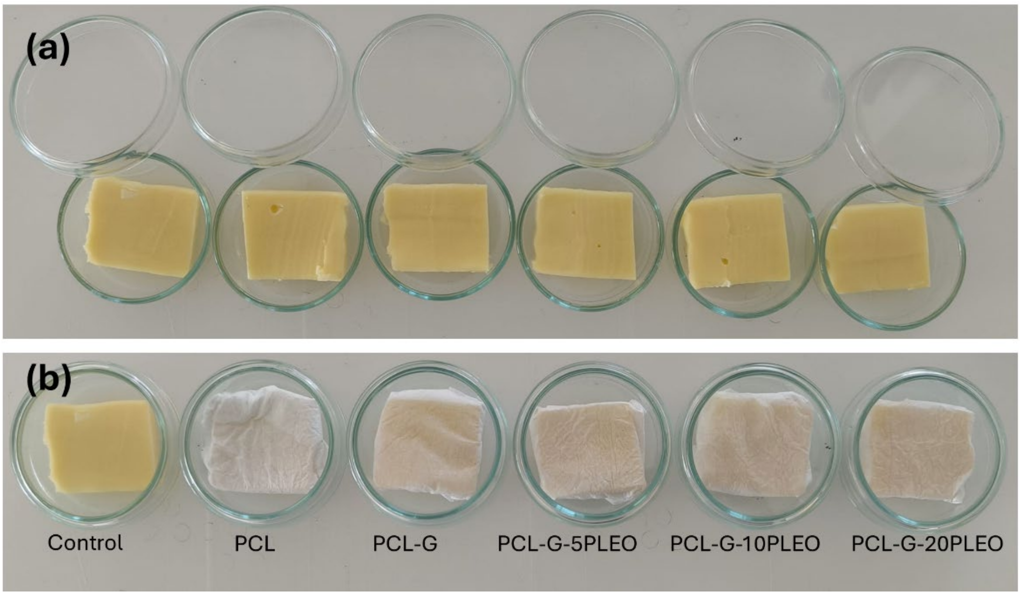

Supplementary Figure S1. Cheese samples before and after application of nanofibrous mats. (a) Untreated cheese samples prior to wrapping. (b) Cheese samples after wrapping with nanofibrous mats including the following formulations: Control, PCL, PCL-G, PCL-G-5PLEO, PCL-G-10PLEO, and PCL-G-20PLEO. Each cheese piece was wrapped with a double layer of the corresponding nanofiber film, except the control sample, which remained unwrapped.
